# The Endocannabinoid System, an Underexploited and Promising Niche for the Pharmacological Treatment of Obesity and Metabolic Diseases

**DOI:** 10.3390/nu14030421

**Published:** 2022-01-18

**Authors:** Javier Gómez-Ambrosi

**Affiliations:** 1 Metabolic Research Laboratory, Clínica Universidad de Navarra, 31008 Pamplona, Spain; jagomez@unav.es; 2 Centro de Investigación Biomédica en Red-Fisiopatología de la Obesidad y Nutrición (CIBEROBN), Instituto de Salud Carlos III, 31008 Pamplona, Spain; 3 Obesity and Adipobiology Group, Instituto de Investigación Sanitaria de Navarra (IdiSNA), 31008 Pamplona, Spain

Obesity represents the most prevalent metabolic disease in the world at present, posing an important public health challenge. This circumstance has led to a better understanding of the mechanisms that control body weight and energy homeostasis. Obesity elevates the risk of developing type 2 diabetes, cardiovascular disease, non-alcoholic fatty liver disease, and certain types of cancer, among other conditions, which translates into a shortened life expectancy [1].

The pillars of the therapeutic approach for patients living with obesity are a reduction in caloric intake and an increase in energy expenditure. Even a modest weight loss is associated with notable improvements in obesity-associated comorbidities. However, when lifestyle modification fails, before resorting to bariatric surgery, pharmacological intervention should be considered as an important alternative or adjunct therapy for weight loss. In this sense, achieving weight normalization by undergoing long-term drug therapy with sufficient tolerability and safety remained an unattainable challenge until recently [2].

The endocannabinoid (eCB) system is an endogenous signaling pathway formed by lipid-derived endogenous mediators (endocannabinoids), their receptors (cannabinoid type 1 and type 2 (CB_1_ and CB_2_)), and the enzymes involved in their synthesis and degradation [3]. The endocannabinoid receptor CB_1_R in the brain is involved in the regulation of food intake and energy homeostasis. Selective antagonism or inverse agonism of this receptor in the brain using drugs such as rimonabant reduced appetite, enhanced thermogenesis and diminished lipogenesis in several human trials, but important psychiatric side-effects precluded its approval for the treatment of obesity by the FDA and forced its withdrawal from the European markets [2]. However, the peripheral antagonism of this receptor with drugs that do not cross the blood–brain barrier has been proposed as an interesting strategy for the management of many obesity-associated metabolic alterations [4].

In an excellent review article published in *Nutrients*, DiPatrizio summarizes the available evidence, suggesting that the eCB in the upper gastrointestinal tract is involved in the control of the gut–brain neurotransmission through the vagus nerve regulating food intake [5]. The author describes the indirect and direct mechanisms controlling the intake of palatable food and the impact of diet-induced obesity (DIO) on those signaling pathways. Tasting dietary fats, specifically unsaturated fats, trigger the production of eCBs, 2-arachidonoyl-sn-glycerol (2-AG) and anandamide in the upper small-intestinal epithelium, eliciting positive feedback to the brain that promotes the intake of fatty foods in rodents [5,6,7]. Humans prefer fatty and sweet foods, and their consumption increases blood levels of eCBs, which are also increased in patients with obesity. However, whether circulating eCBs influence dietary choices in humans remains to be determined [5].

Among the indirect mechanisms involved in the regulation of food intake by gut eCB, DiPatrizio extensively details the nutrient-induced blocking of the release of cholecystokinin (CCK), a satiating peptide, after CB_1_R activation in rodents. The ability of nutrients to induce CCK release is restored after the pharmacological inhibition of peripheral CB_1_Rs in mice with DIO, reducing food intake [5]. Moreover, studies suggest that CB_1_R is also involved in the production of ghrelin, an orexigenic peptide, by the stomach. Future studies will confirm if the regulation of the release of peptides involved in food intake control by eCB is also altered in patients with obesity and the specific intracellular signaling pathways that are implicated.

Food-related signals released from the gut may directly regulate the activity of vagal afferent neurons through the activation of CB_1_Rs. Moreover, vagal efferent neurons drive the production of 2-AG and local CB_1_Rs activation in the intestine during fasting via the activation of parasympathetic m3 acetylcholine receptors. In addition, there are further interactions between the eCB system and sympathetic neurotransmission in the control of food intake and, in this sense, the review describes how this interaction is important in the metabolic benefits of Roux-en-Y gastric bypass, a type of metabolic surgery, in mice [5,8].

Rodent and human studies evidence that the eCB system is a relevant branch of the gut–brain axis that participates in the regulation of food intake and is altered in obesity [5]. As has been mentioned above, the use of CB_1_R antagonists or inverse agonists, such as rimonabant, exert anti-obesity actions but have undesirable psychiatric side-effects. In recent years, it has become evident that the peripheral eCB system plays an important role in both food intake regulation [9] and glucose and lipid homeostasis, with major actions in the liver and adipose tissue [3,10], thereby representing an emerging target for the development of new pharmacologic tools to fight against metabolic diseases. In addition, DiPatrizio also highlights the potential of new CB_1_R antagonists with low brain penetrance, which may be effective and safe therapeutic options for the treatment of obesity and related conditions [4,11]. Finally, the author underlines the importance of future studies aiming to disentangle the molecular and cellular mechanisms in the interactions between the eCB system and the gut microbiota on energy homeostasis [5,12].
